# Hypoglycemia unawareness identified by continuous glucose monitoring system is frequent in outpatients with type 2 diabetes without receiving intensive therapeutic interventions

**DOI:** 10.1186/s13098-022-00959-x

**Published:** 2022-11-28

**Authors:** Bingkun Huang, Qiuhui Jiang, Ting Wu, Qingbao Shen, Wengui Wang, Shoubi Wang, Yinxiang Huang, Shunhua Wang, Peiying Huang, Mingzhu Lin, Xiulin Shi, Xuejun Li

**Affiliations:** 1 Department of Endocrinology and Diabetes, Xiamen Diabetes Institute, Xiamen Clinical Medical Center for Endocrine and Metabolic Diseases, Xiamen Diabetes Prevention and Treatment Center, Fujian Key Laboratory of Diabetes Translational Medicine, The First Affiliated Hospital of Xiamen University, School of Medicine, Xiamen University, Xiamen, China; 2 The Third Clinical Medical College of Fujian Medical University, Fujian, China; 3 Fujian Provincial Key Laboratory of Ophthalmology and Visual Science, Eye Institute of Xiamen University, School of Medicine, Xiamen University, Xiamen, China

**Keywords:** Hypoglycemia unawareness, Asymptomatic hypoglycemia, Continuous glucose monitoring system, Type 2 diabetes

## Abstract

**Background:**

Patients with diabetes are prone to asymptomatic hypoglycemia (AH) due to diminished ability to perceive the onset of hypoglycemia. However, the actual prevalence and influencing factors of AH in outpatients with type 2 diabetes (T2DM) have not been well investigated.

**Methods:**

A total of 351 outpatients with T2DM underwent glucose monitoring by continuous glucose monitoring system (CGMS) for consecutive 72 h without changing their lifestyle and treatment regimens. Hypoglycemia is defined as a blood glucose level less than 3.9 mmol/L, which was further divided into Level 1 hypoglycemia (blood glucose 3.0–3.9 mmol/L) and Level 2 hypoglycemia (blood glucose < 3.0 mmol/L). Univariate and multivariate logistic regression analyses were used to determine the possible risk factors of AH.

**Results:**

In all 351 subjects studied, 137 outpatients (39.0%) were captured AH events, in which Level 1 AH and Level 2 AH accounted for 61.3% and 38.7%, respectively. 85 (62.0%) of the AH patients experienced nocturnal asymptomatic hypoglycemia (NAH) and 25 (18.2%) exclusively NAH. Multivariate logistic regression analysis demonstrated that patients with younger age, lower hemoglobin A1c (HbA1c), and higher systolic blood pressure (SBP) levels were associated with increased risk of AH. While after further grading of AH, male sex and Dipeptidylpeptidase-4 inhibitors (DPP4i) regime were shown to be associated with lower risk of Level 2 AH.

**Conclusions:**

Hypoglycemia unawareness could be frequently observed at either daytime or nighttime, although NAH was more common, in outpatients with T2DM. Relative relax HbA1c targets should be considered for patients who are prone to AH.

**Supplementary Information:**

The online version contains supplementary material available at 10.1186/s13098-022-00959-x.

## Introduction

Hypoglycemia is well-recognized as one of the most severe complications and therapy-limited factors of patients with diabetes (reviewed by Amiel SA et al*.*) [1]. It has been reported that hypoglycemia could not only cause unpleasant symptoms, impaired cognitive function, diminished productivity, but also result in long-term chronic vascular complications of diabetes and affect the lifespan [2]. Theoretically, when hypoglycemia occurs, counter-regulatory systems are normally activated and patients with diabetes tend to develop the characteristic neuroglycopenic and adrenergic symptoms of hypoglycemia, which is regarded as a protective mechanism [3, 4]. Whereas, in clinical practice, there are quite a few patients experiencing hypoglycemia episodes without any sensed symptoms, which is defined as asymptomatic hypoglycemia (AH). AH accounted for 36% of the hypoglycemia events that occurred while subjects were awake, according to the Diabetes Control and Complications Trial (DCCT) [4]. Studies by Gold et al*.* [5] and Akram et al*.* [6] have revealed that being the most critical risk factor for severe hypoglycemia, impaired perception of hypoglycemia could lead to a six-fold increase in the frequency of severe hypoglycemia, thus significantly increasing the potential risks of severe adverse events [7]. Additionally, antecedent hypoglycemia attacks could shift glycemic thresholds for autonomic and symptomatic responses to lower plasma glucose concentrations and further aggravate impaired awareness of hypoglycemia, with evidence that avoiding such exposure could restore consciousness and terminate the vicious cycle of recurrent hypoglycemia [8–10]. Nocturnal asymptomatic hypoglycemia (NAH), a significant problem noted in the AH population, is more likely to be ignored because patients do not monitor their blood glucose (BG) while sleeping, which accounted for 55% of severe hypoglycemic events in the DCCT [4]. It has been reported that NAH could cause life-threatening events, such as “dead in bed” syndrome [10, 11].

The continuous glucose monitoring system (CGMS) is rapidly improving diabetes management and has been proved to be superior to daily self-monitoring blood glucose (SMBG) in the detection of hypoglycemia and in improving glycemic control in T2DM individuals [12]. By using CGMS, Chico et al*.* [13] detected a substantially high proportion of AH, including 62.5% of T1DM patients and 46.6% of T2DM patients. Data recorded via 72-h CGMS [14] revealed that the incidence of NAH in T2DM patients was twofold higher than that of daytime hypoglycemia. Similarly, a 1-year prospective study of 350 insulin-dependent children with diabetes revealed that 56% of severe hypoglycemic episodes occurred at night [15]. McNally et al*.* [14, 16] noted the unrecognized hypoglycemia in patients with T2DM and took necessary treatment adjustments, significantly reducing the frequency of hypoglycemia. Thereby, concerning the reliability and accuracy, it is considerable for CGMS to be brought into the routine management of T2DM.

Rates and influencing factors of AH have been widely reported in type 1 diabetes (T1DM) [17–19] and T2DM [20, 21] patients with intensive treatment who are prone to hypoglycemia. It is generally assumed that the risk of developing hypoglycemia in daily diabetes outpatients is relatively lower because their medical conditions are relatively stable. A small sample size research (31 participants) reported that 83.1% (69 out of 83) hypoglycemia episodes were unrecognized in outpatients with T2DM [16]. Another study in 222 outpatients with T2DM demonstrated that the AH was frequently seen [22]. Given the high prevalence, potential and prolonged harms, and insidious onset of AH, estimating the actual rate and promptly identifying episodes of AH have important implications for diabetes care. In view of the insufficient attention to T2DM outpatients without receiving intensive therapeutic interventions, our study enrolled such an entity and aimed to determine the influencing factors of AH in T2DM outpatients via CGMS.

## Research design and methods

### Study design

This study included 351 outpatients with T2DM in the First Affiliated Hospital of Xiamen University from January 2018 to March 2022. Exclusions included children (< 18 years), pregnant women, and patients who had the following: perioperative, bleeding disorders, cognitive dysfunction, alcohol abuse, taking beta-blockers or selective serotonin reuptake inhibitors (SSRIs), and incomplete data due to dropped probe. The studies involving human participants were reviewed and approved by The First Affiliated Hospital of Xiamen University. Enrolled patients completed the informed consent and wore the CGMS device at the outpatient clinic. In order to reflect the effectiveness and safety of the current treatment regimen of the subjects, we did not ask them to change their previous diet and exercise habits during the CGM test. Thus, they went home to lead a normal life for three consecutive days. Clinical data of baseline characteristics, such as Glycated hemoglobin A1c (HbA1c), BMI (calculated as weight in kilograms divided by the square of height in meters), diabetes duration, age, diabetes medications, complications, and diabetic complications, were collected. CGM (continuous glucose monitoring) test ran for three days on average, and data were obtained through iPro™ version 2 (Medtronic Minimed, MMT-7745WW). The glucose value is recorded every 5 min, for a total of 288 readings a day. Before the CGM test, we provided unified blood glucose meters (Contour Plus Blood Glucose Meter, 7600P) to each patient and instructed them to provide at least four self-monitoring blood glucose (SMBG) tests per day. Besides, notebooks were provided to each patient to record the frequency and time of suspicious hypoglycemia if they had symptoms such as hunger, palpitations, tremulousness, sweats, and dizziness. After three days of monitoring, patients returned to the outpatient clinic, where their SMBG metrics were imputed into the system and the CGM data was downloaded. The CGM data recorded in this article included the glucose standard deviation (SD), mean blood glucose (MBG), the mean amplitude of glycemic excursions (MAGE), time in range (TIR, 3.9–10.0 mmol/L), time below range (TBR, < 3.9 mmol/L), time above range (TAR, > 10.0 mmol/L), the number of hypoglycemic events, and the corresponding duration of hypoglycemic episodes.

### Definition of hypoglycemia

According to the definition of the International Hypoglycemia Study Group [1], hypoglycemia is defined as a blood glucose level less than 3.9 mmol/L, which was further divided into Level 1 hypoglycemia (BG 3.0–3.9 mmol/L) and Level 2 hypoglycemia (BG < 3.0 mmol/L) according to the minimum blood glucose value. AH was defined as BG < 3.9 mmol/L without any sensed symptoms. Daytime asymptomatic hypoglycemia (DAH) was defined as BG < 3.9 mmol/L during 6:00–24:00. NAH was defined as BG < 3.9 mmol/L during 0:00–6:00. A single hypoglycemic episode was defined as BG below 3.9 mmol/L for at least 15 min [23]. Two separate hypoglycemic events were defined when the time interval between two hypoglycemic events was equal to or greater than 30 min.

### Statistical analysis

Preliminary statistical analyses included descriptive statistics and assessment of distributions. Variables are presented as the mean ± standard deviation, median with interquartile range or percentages where appropriate. The statistical significance of differences between different groups was assessed by *t* test or Kruskal–Wallis test for continuous variables and by Chi-square test for categorical variables. The univariate and multivariate logistic regression analyses were used to determine clinical predictors of hypoglycemia. Those found to be significant in univariate models and some characteristics selected a priori were adjusted for in multivariate logistic regression models. Statistical analyses were performed with SAS version 9.3 statistical software. All the statistical tests were two-sided. P-value < 0.05 was considered statistically significant.

## Results

### Characteristics and CGM data of the enrolled outpatients

The characteristics of the total 351 outpatients with T2DM were shown in Table 1. There were 195 (55.6%) men and 156 (44.4%) women with an average age of 55.4 ± 14.4 years. The average systolic blood pressure (SBP) was 125.5 ± 15.9 mmHg, and the average diastolic blood pressure (DBP) was 75.9 ± 10.0 mmHg. The mean BMI was 22.9 ± 3.1 kg/m^2^. The median duration of diabetes was 6.0 years, and the mean HbA1c was 7.1 ± 1.5% (54 ± 16 mmol/mol). According to 3-day CGM data and self-reports of the subjects, none experienced symptomatic hypoglycemia. Overall, AH events were captured in 137 outpatients (39.0%), in whom Level 1 and Level 2 AH accounted for 61.3% and 38.7%, respectively. 112 (31.9%) of the AH population occurred during the daytime, and 85 (24.2%) experienced NAH. Hypoglycemia episodes that occurred exclusively at night accounted for 18.2% of the AH population and 29.4% of the NAH population. The number of hypoglycemic events among those with daytime asymptomatic hypoglycemia (DAH), nocturnal asymptomatic hypoglycemia (NAH), asymptomatic hypoglycemia occurred exclusively in the daytime (DAH*), asymptomatic hypoglycemia occurred exclusively at night (NAH*), and asymptomatic hypoglycemia occurred in both daytime and nighttime were 3.0 (1.0, 10.0), 6.0 (3.0, 12.0), 1.0 (1.0, 4.0), 1.0 (0.0,4.0), and 8.0 (3.0, 14.5), respectively. The Total TIR was 84.9 (66.7, 96.0). The MAGE, SD, and MBG were 4.2 ± 2.2 mmol/L, 1.9 ± 0.9 mmol/L, and 8.2 ± 2.1 mmol/L, respectively.

**Table 1:** Clinical characteristics and CGM data of the enrolled outpatients

Characteristics	n = 351
Sex	
Male	195 (55.6)
Female	156 (44.4)
Age, years	55.4 ± 14.4
SBP, mmHg	125.5 ± 15.9
DBP, mmHg	75.9 ± 10.0
BMI, kg/m^2^	22.9 ± 3.1
Duration, years	6.0 (2.0, 11.0)
HbA1c, %	7.1 ± 1.5
HbA1c, mmol/mol	54 ± 16
C-P, ng/mL	1.4 (0.6,2.0)
Comorbidity	
Hypertension	45 (12.8)
Hyperlipidemia	59 (16.8)
Fatty liver	19 (5.4)
CCVD	37 (10.5)
Medication	
Metformin	97 (27.6)
DPP-4i	57 (16.2)
SGLT-2i	15 (4.3)
α-GI	65 (18.5)
GLP-1ra	6 (1.7)
TZD	9 (2.6)
SU	78 (22.2)
Insulin therapy	137 (39.0)
Long-acting insulin	78 (22.2)
Premixed insulin	36 (10.3)
Short-acting insulin	58 (16.5)
Complication	
DR	54 (15.4)
DPN	45 (12.8)
DPVD	17 (4.8)
DN	17 (4.8)
DF	2 (0.6)
CGM data	
AH (%)	137 (39.0)
Total TIR, %	84.9 (66.7,96.0)
Nighttime TIR, %	96.8 (76.0, 100.0)
Daytime TIR, %	81.9 (61.6,95.6)
Total TBR, %	2.1 (0.8,5.8)
Nighttime TBR, %	1.8 (0.0, 10.2)
Daytime TBR, %	1.1 (0.2,3.7)
Total TAR, %	12.7 (2.2,32.1)
Nighttime TAR, %	0.0 (0.0, 17.1)
Daytime TAR, %	16.2 (2.9,37.6)
Level 1 AH (%)	84 (23.9)
Total TIR, %	89.4 (77.6,97.2)
Nighttime TIR, %	96.3 (80.4, 100.0)
Daytime TIR, %	88.9 (72.6,97.0)
Total TBR, %	1.0 (0.6,2.6)
Nighttime TBR, %	0.1 (0.0,6.3)
Daytime TBR, %	0.8 (0.2, 1.5)
Total TAR, %	6.2 (1.0,23.2)
Nighttime TAR, %	0.0 (0.0,2.2)
Daytime TAR, %	8.2 (1.3,26.8)
Level 2 AH (%)	53 (15.1)
Total TIR, %	74.8 (67.0,85.7)
Nighttime TIR, %	81.0 (65.5,95.5)
Daytime TIR, %	74.3 (63.3–85.3)
Total TBR, %	4.8 (2.5,8.6)
Nighttime TBR, %	4.6 (1.3, 18.2)
Daytime TBR, %	3.4 (1.0,7.2)
Total TAR, %	18.3 (3.9,28.5)
Nighttime TAR, %	0.7 (0.0, 19.0)
Daytime TAR, %	20.6 (5.3,33.8)
DAH (%)	112 (31.9)
Hypoglycemic events	3.0 (1.0, 10.0)
Hypoglycemic duration, min	27.0 (11.5,94.5)
NAH (%)	85 (24.2)
Hypoglycemic events	6.0 (3.0, 12.0)
Hypoglycemic duration, min	51.0 (23.0, 103.0)
DAH*(%)	52 (14.8)
Hypoglycemic events	1.0 (1.0, 3.0)
Hypoglycemic duration, min	12.0 (4.5, 25.0)
NAH*(%)	25 (7.1)
Hypoglycemic events	1.0 (0.0, 4.0)
Hypoglycemic duration, min	6.0 (0.0, 37.0)
Both DAH and NAH (%)	60 (17.1)
Hypoglycemic events	8.0 (3.0, 14.5)
Hypoglycemic duration, min	68.0 (27.0,135.0)
MAGE, mmol/L	4.2 ± 2.2
SD, mmol/L	1.9 ± 0.9
MBG, mmol/L	8.2 ± 2.1

Data were present as Mean ± SD or Median (25th percentile, 75th percentile); *SBP* systolic blood pressure, *DBP* diastolic blood pressure, *BMI* body mass index, *HbA1c* glycated hemoglobin A1c, *C-P* C-Peptide, *CCVD* cardio-cerebral vascular disease, *DPP-4i* Dipeptidylpeptidase-4 inhibitors, *SGLT-2i* sodium–glucose cotransporter 2 inhibitors, *α-GI* alpha-glucosidase inhibitors, *GLP-1RA* glucagon-like peptide-1 receptor agonists, *TZD* thiazolidinedione, *SU* sulfonylurea, *DR* diabetic retinopathy, *DPN* diabetic peripheral neuropathy, *DPVD* diabetic peripheral vascular disease, *DN* diabetic nephropathy, *DF* diabetic foot, *CGM* continuous glucose monitoring, *Level 1 AH* BG 3.0–3.9 mmol/L, *Level 2 AH* BG < 3.0 mmol/L, *Total TIR* total time in range (3.9–10.0 mmol/L), *Nighttime TIR* time in range (3.9–10.0 mmol/L) during 0:00–6:00, *Daytime TIR* time in range (3.9–10.0 mmol/L) during 6:00–24:00, *Total TBR* total time below range (< 3.9 mmol/L), *Nighttime TBR* time below range (< 3.9 mmol/L) during 0:00–6:00, *Daytime TBR* time below range (< 3.9 mmol/L) during 6:00–24:00, *Total TAR* total time above range (> 10.0 mmol/L), *Nighttime TAR* time above range (> 10.0 mmol/L) during 0:00–6:00, *Daytime TAR* time above range (> 10.0 mmol/L) during 6:00–24:00, *DAH* daytime asymptomatic hypoglycemia (defined as 6:00–24:00 blood glucose < 3.9 mmol/L), DAH* asymptomatic hypoglycemia occurred exclusively in the daytime, *NAH* nocturnal asymptomatic hypoglycemia (defined as 0:00–6:00 blood glucose < 3.9 mmol/L), NAH* asymptomatic hypoglycemia occurred exclusively at night, *both DAH and NAH* asymptomatic hypoglycemia episodes occurred in both daytime and nighttime, *MAGE* mean amplitude of plasma glucose excursion, *SD* glucose standard deviation, *MBG* mean blood glucose

### Comparisons between the Overall AH group and the non-AH group and assessment of influencing factors for Overall AH

In Table 2, compared to outpatients without AH, outpatients in the AH group showed younger age (52.1 ± 14.6 years old vs. 55.9 ± 14.1 years old, P < 0.05) and lower HbA1c (6.8 ± 1.3% vs. 7.2 ± 1.6%, P < 0.05). According to the data of CGM, glucose SD (2.2 ± 1.0 mmol/L vs. 1.7 ± 0.9 mmol/L, P < 0.001) and MAGE (4.8 ± 2.3 mmol/L vs. 3.8 ± 2.0 mmol/L, P < 0.001) in the AH group were significantly higher than those without AH, while MBG (7.5 ± 1.4 mmol/L vs. 8.7 ± 2.2 mmol/L, P < 0.001) was significantly lower. Nighttime TIR in the AH group was significantly lower than that in the non-AH group [92.0 (75.8 ,100.0) vs. 100.0 (76.4, 100.0), P = 0.002]. Patients had AH episodes exhibited lower Total TAR [92.0 (1.4,25.0) vs. 14.7 (3.0,38.7), P < 0.01], lower Daytime TAR [12.3 (2.0,31.6) vs. 18.5 (4.1,44.6), P < 0.01], and lower Nighttime TAR [0.0 (0.0, 10.0) vs. 0.0 (0.0,23.6), P < 0.05]. In order to determine the influencing factors of AH, we conducted a series of analyses. After controlling for all factors identified through univariate analyses (Additional file 1: Table S1), multivariate analysis (Table 3) demonstrated that patients with younger age [0.975 (0.955–0.997), P = 0.023], lower HbA1c levels [0.759 (0.620–0.929), P = 0.008], and higher SBP [1.024 (1.002–1.047), P = 0.033] had a significantly higher risk of experiencing overall AH episodes.

**Table2:** Clinical characteristics and CGM data of non-AH and AH patients

	Non-AH(n = 214)	AH (n = 137)		
		Overall AH (n = 137)	Level 1 AH (n = 84)	Level 2 AH (n = 53)
Sex				
Male	124 (57.9)	71 (51.8)	50 (59.5)	21 (39.6) *
Female	90 (42.1)	66 (48.2)	34 (40.5)	32 (60.4)
Age, years	55.9 ± 14.1	52.1 ± 14.6*	55.3 ± 12.9	47.0 ± 15.8***
SBP, mmHg	125.2 ± 15.9	126.0 ± 16.0	128.1 ± 16.0	122.5 ± 15.7
DBP, mmHg	76.3 ± 10.6	75.3 ± 9.0	76.0 ± 8.3	74.2 ± 10.0
BMI, kg/m^2^	23.1 ± 3.0	22.5 ± 3.1	22.4 ± 2.9	22.8 ± 3.4
Duration, years	6.0 (2.0, 11.0)	6.0 (2.3, 11.0)	6.0 (3.0,11.0)	6.0 (2.0,14.0)
HbA1c, %	7.2 ± 1.6	6.8 ± 1.3*	6.7 ± 1.2**	7.0 ± 1.6
HbA1c, mmol/mol	56 ± 17	51 ± 15*	50 ± 13**	53 ± 17
C-P, ng/mL	1.5 (0.7, 2.2)	1.2 (0.4, 1.9)	1.5 (1.1,2.6)	0.5 (0.1,1.6) **
Comorbidity				
Hypertension	27 (12.6)	18 (13.1)	14 (16.7)	4 (7.5)
Hyperlipidemia	36 (16.8)	23 (16.8)	16 (19.0)	7 (13.2)
Medication				
Metformin	63 (29.4)	34 (24.8)	25 (29.8)	9 (17.0)
DPP-4i	36 (16.8)	21 (15.3)	20 (23.8)	1 (1.9) **
α-GI	42 (19.6)	23 (16.8)	18 (21.4)	5 (9.4)
SU	46 (21.5)	32 (23.4)	23 (27.4)	9 (17.0)
Insulin	79 (36.9)	58 (42.3)	30 (35.7)	28 (52.8) *
Complication				
DR	32 (15.0)	22 (16.1)	11 (13.1)	11 (20.8)
DPN	31 (14.5)	14 (10.2)	8 (9.5)	6 (11.3)
DPVD	11 (5.1)	6 (4.4)	3 (3.6)	3 (5.7)
DN	11 (5.1)	6 (4.4)	4 (4.8)	2 (3.8)
DF	2 (0.9)	0 (0.0)	0 (0.0)	0 (0.0)
CGM data				
MAGE, mmol/L	3.8 ± 2.0	4.8 ± 2.3***	4.4 ± 2.0*	5.2 ± 2.5***
SD, mmol/L	1.7 ± 0.9	2.2 ± 1.0***	1.9 ± 0.9	2.5 ± 1.0***
MBG, mmol/L	8.7 ± 2.2	7.5 ± 1.4***	7.4 ± 1.3***	7.6 ± 1.6***
Total TIR, %	85.3 (61.3, 96.9)	84.8 (70.8, 95.4)	89.4 (79.7, 96.8)	78.1 (67.0, 86.0) *
Nighttime TIR, %	100.0(76.4, 100.0)	92.0 (75.8, 100.0) **	96.8 (80.6, 100.0)	81.5 (65.5, 95.8) ***
Daytime TIR, %	81.1 (55.4.95.9)	84.7 (67.8, 95.3)	89.3 (73.7, 96.8) *	76.0 (63.3, 85.9)
Total TAR, %	14.7 (3.0, 38.7)	9.2 (1.4, 25.0) **	6.2 (1.2, 20.1) **	18.2 (3.9, 28.6)
Nighttime TAR, %	0.0 (0.0, 23.6)	0.0 (0.0, 10.0) *	0.0 (0.0, 2.5) **	0.0 (0.0, 19.9)
Daytime TAR, %	18.5 (4.1, 44.6)	12.3 (2.0, 31.6) **	8.2 (1.6, 26.3) **	19.0 (5.3, 34.8)

Data were present as Mean ± SD or Median (25th percentile, 75th percentile). *SBP* systolic blood pressure, *DBP* diastolic blood pressure, *BMI* body mass index, *HbA1c* glycated hemoglobin A1c, *C-P* C-Peptide, *DPP-4i* Dipeptidylpeptidase-4 inhibitors, *α-GI* alpha-glucosidase inhibitors, *SU* sulfonylurea, *DR* diabetic retinopathy, *DPN* diabetic peripheral neuropathy, *DPVD* diabetic peripheral vascular disease, *DN* diabetic nephropathy, *DF* diabetic foot, *CGM* continuous glucose monitoring, *Level 1 AH* BG 3.0–3.9 mmol/L, *Level 2 AH* BG < 3.0 mmol/L, *DAH* daytime asymptomatic hypoglycemia (defined as 6:00–24:00 blood glucose < 3.9 mmol/L), DAH* asymptomatic hypoglycemia occurred exclusively in the daytime, *NAH* nocturnal asymptomatic hypoglycemia (defined as 0:00–6:00 blood glucose < 3.9 mmol/L), NAH* asymptomatic hypoglycemia occurred exclusively at night, *both DAH and NAH* asymptomatic hypoglycemia episodes occurred in both daytime and nighttime, *MAGE* mean amplitude of plasma glucose excursion, *SD* glucose standard deviation, *MBG* mean blood glucose, *TBR* time below range (< 3.9 mmol/L), *Total TIR* time in range (3.9–10.0 mmol/L), *Nighttime TIR* time in range (3.9–10.0 mmol/L) during 0:00–6:00, *Daytime TIR* time in range (3.9–10.0 mmol/L) during 6:00–24:00, *Total TAR* time above range (> 10.0 mmol/L), *Nighttime TAR* time above range (> 10.0 mmol/L) during 0:00–6:00, *Daytime TAR* time above range (> 10.0 mmol/L) during 6:00–24:00

* P < 0.05 for the specified comparison of AH group and non-AH group; ** P < 0.01 for the specified comparison of AH group and non-AH group; *** P < 0.001 for the specified comparison of AH group and non-AH group

**Table3:** Predictors of AH, Level 1 AH, and Level 2 AH on the basis of logistic regression models

Multivariate analysis						
Predictor	AH		Level 1 AH		Level 2 AH	
	OR (95%Cl)	P	OR (95%Cl)	P	OR (95%Cl)	P
Sex	0.822 (0.494–1.368)	0.450	1.610 (0.886–2.923)	0.118	0.391 (0.191–0.799)	0.010
Age	0.975 (0.955–0.997)	0.023	0.996 (0.971–1.021)	0.746	0.962 (0.936–0.990)	0.008
Duration	1.016 (0.969–1.066)	0.506	0.986 (0.931–1.044)	0.626	1.041 (0.978–1.108)	0.211
Metformin	0.816 (0.440–1.513)	0.519	0.943 (0.492–1.883)	0.867	0.715 (0.274–1.862)	0.492
DPP-4i	0.937 (0.460–1.908)	0.858	2.011 (0.927–4.361)	0.077	0.110 (0.014–0.855)	0.035
α-GI	0.993 (0.517–1.906)	0.982	1.272 (0.627–2.577)	0.505	0.647 (0.225–1.859)	0.419
SU	1.218 (0.635–2.334)	0.553	1.112 (0.545–2.272)	0.770	1.361 (0.511–3.624)	0.538
HbA1c	0.759 (0.620–0.929)	0.008	0.683 (0.527–0.887)	0.004	0.940 (0.727–1.216)	0.639
insulin	1.261 (0.682–2.333)	0.460	1.197 (0.593–2.414)	0.616	1.198 (0.510–2.813)	0.679
SBP	1.024 (1.002–1.047)	0.033	1.033 (1.008–1.059)	0.010	1.000 (0.969–1.032)	0.999
DBP	0.981 (0.950–1.013)	0.238	0.980 (0.946–1.016)	0.272	0.991 (0.946–1.038)	0.692
BMI	0.961 (0.882–1.047)	0.365	0.920 (0.831–1.018)	0.108	1.063 (0.944–1.198)	0.310

*OR* odds ratio, *CI* confidence interval, *DPP-4i* Dipeptidylpeptidase-4 inhibitors, *α-GI* alpha-glucosidase inhibitors, *SU* sulfonylurea, *HbA1c* glycated hemoglobin A1c, *SBP* systolic blood pressure, *DBP* diastolic blood pressure, *BMI* body mass index

### Comparisons between the Level 1 AH group and the non-AH group and assessment of influencing factors for Level 1 AH

As for Level 1 AH, patients with lower HbA1c exhibited a higher prevalence of AH (6.7 ± 1.2% vs. 7.2 ± 1.6%, P < 0.01). The MBG of the AH group was substantially lower (7.4 ± 1.3 vs. 8.7 ± 2.2, P < 0.001) while the MAGE was higher (4.4 ± 2.0 vs. 3.8 ± 2.0, P < 0.001). Daytime TIR [89.3 (73.7,96.8) vs. 81.1 (55.4,95.9), P < 0.05] was corelated with Level 1 AH episodes. Patients with lower Total TAR [6.2 (1.2,20.1) vs. 14.7 (3.0,38.7), P < 0.01], lower Nighttime TAR [0.0 (0.0,2.5) vs. 0.0 (0.0,23.6), P < 0.01], and lower Daytime TAR [8.2 (1.6,26.3) vs. 18.5 (4.1,44.6), P < 0.01] were more vulnerable to Level 1 AH. Multivariate analyses (Table 3) controlled for factors associated with high regimen distress in univariate analyses (Additional file 1: Table S1) and found that lower HbA1c levels [0.683 (0.527–0.887), P = 0.004] and higher SBP [1.033 (1.008–1.059), P = 0.010] were also associated with Level 1 AH.

### Comparisons between the Level 2 AH group and the non-AH group and assessment of influencing factors for Level 2 AH

There was greater percentage of female in the Level 2 AH group (60.4% vs. 39.6%, P < 0.05). The age (47.0 ± 15.8 vs. 55.9 ± 14.1, P < 0.001) of the Level 2 AH group was significantly lower and the C-Peptide (C-P) [0.5 (0.1,1.6) vs. 1.5 (0.7, 2.2), P < 0.01] level was lower. Significantly statistical difference was observed in the use of Dipeptidylpeptidase-4 inhibitors (DPP4i) (1.9% vs. 16.8%, P < 0.01) and insulin regime (52.8% vs. 36.9%, P < 0.05) between Level 2 AH group and non-AH group. The MAGE (5.2 ± 2.5 mmol/L vs. 3.8 ± 2.0 mmol/L, P < 0.001) and glucose SD (2.5 ± 1.0 mmol/L vs. 1.7 ± 0.9 mmol/L, P < 0.001) in the AH group were significantly higher than those without AH, while MBG (7.6 ± 1.6 mmol/L vs. 8.7 ± 2.2 mmol/L, P < 0.001) was significantly lower. There were significant statistical differences in Total TIR [78.1 (67.0,86.0) vs. 85.3 (61.3,96.9), P < 0.05] and Nighttime TIR [81.5 (65.5,95.8) vs. 100.0 (76.4,100.0), P < 0.001] between two groups. After controlling for all factors identified through univariate analyses (Additional file 1: Table S1), multivariate analyses found that female sex [0.391 (0.191–0.799), P = 0.010] and younger age [0.962 (0.936–0.990), P = 0.008] were independent predictors of Level 2 AH episodes. In addition, patients treated with DPP4i tended to have lower risk of Level 2 AH [0.110 (0.014–0.855), P = 0.035]. (Table 3).

## Discussion

In the current study, 137 (39.0%) of the total 351 outpatients with T2DM experienced AH episodes, in which Level 1 AH and Level 2 AH accounted for 61.3% and 38.7%, respectively. 85 (62.0%) of the AH patients experienced NAH and 25 (18.2%) exclusively NAH. Multivariate logistic regression analysis demonstrated that patients with younger age, lower HbA1c, and higher SBP levels were significantly associated with increased risk of AH. While after further grading of AH, male sex and DPP4i regime were shown to be associated with lower risk of Level 2 AH.

Previously, Chico et al*.* [13] detected unrecognized hypoglycemias in 46.6% (14 out of 30) of the type 2 patients with diabetes by using CGMS and noticed that 73.7% of all events occurred at night, tremendously provoking attention from academia. Further, Gehlaut et al*.* [24] applied CGMS on 108 non-hospitalized patients with T2DM to detect the episodes of hypoglycemia. During the five-day study, 53 (49.1%) participants were captured of hypoglycemia by CGMS, but only 24.5% (13 out of 53) had self-reported symptoms, while the majority (75%) of patients were unaware of any hypoglycemia episodes. The prevalence of NAH was 73.6% (37 out of 53), and hypoglycemia occurred only at night was 20.8% (14 out of 53). In our study, none of the participants reported symptomatic hypoglycemia, and 39.0% of the target patients experienced AH events, which was in parallel with previous studies. Notably, all patients experienced hypoglycemic episodes claimed they had no symptoms of hypoglycemia. We speculate that the heterogeneity of symptom response in diabetic individuals could took into consideration and selective bias might play a role. It is also possible that the patients were asymptomatic due to recurrent episodes of hypoglycemia.

Although plenty of studies [12, 17, 22, 23, 25] had indicated that older age, longer diabetes duration, and microvascular complications were independently correlated with increased prevalence of hypoglycemia, in our present study, multivariate logistic regression analysis found no significance of these variables in predicting AH events except for younger age in the Level 2 AH group. Various explanations could be proposed. Firstly, most prior studies were conducted during hospitalization, and they focused merely on the insulin-used population with poor glucose control to investigate the incidence of hypoglycemia. However, the target population of our study was T2DM outpatients whose status was relatively stable with "well-controlled" glucose levels. Differences in samples may contribute to differences of results. Secondly, we used the CGMS to detect the hypoglycemia episodes, which is expected to discover additional unrecognized hypoglycemia events than traditional SMBG.

Previous studies [24, 28] had demonstrated that intensive insulin treatment or sulfonylurea (SU) could predict the episodes of hypoglycemia in T2DM. Our study also showed a higher rate of insulin therapy (52.8% vs. 36.9%) in the Level 2 AH group than in the non-AH group. The univariate analysis (Additional file 1: Table S1) indicated that patients with the use of insulin were associated with higher risk of Level 2 AH (P = 0.018). However, this association did not exist when accounting for other influencing factors in the multiple logistic regression models. This may be due to the substantial effect of the adjusted confounders. Additionally, neither univariate nor multivariate analysis demonstrated the association between SU and the risk of AH. Overall, our study observed that outpatients treated with insulin or SU did not predispose to asymptomatic hypoglycemia, which was consistent with the conclusion of Monnier et al. [22, 29]. This interesting finding reminds us to screen all diabetic patients for a history of hypoglycemia, rather than focusing only on those using insulin or insulin secretagogues.

In our study, the univariate logistic analysis indicated that DPP4i regime was associated with lower risks of Level 1 and Level 2 AH, yet the association between DPP4i and Level 1 AH was substantially attenuated after further adjusting for other confounders. This may stem from the fact that, in our present study, elder age was a protective factor for AH, and the average age of the Level 1 AH group was substantially higher than that of the Level 2 AH group (55.3 ± 12.9 vs. 47.0 ± 15.8). Thus, after controlling for age and other affecting factors, the protective effect of DPP4i in the Level 1 group disappeared. Overall, DPP4i have been proved to be substantially associated with lower risks of hypoglycemia [26]. Further studies are needed to confirm the causal relationship between DPP-4i regime and the risk of AH.

The link between the diagnosis of hypertension and a higher risk of hypoglycemia had been identified in previous studies [27, 28]. In the present study, 12.8% of the subjects had combined hypertension. Multifactorial analysis showed that higher SBP level was predisposed to AH episodes despite the well-controlled blood pressure levels (125.5 ± 75.9 mmHg) of participants. Thus, the role that strict control of SBP may play in patients who were vulnerable to AH needs further attention. Additionally, our preliminary analysis did not observe a significant association between gender and AH, but when further dividing AH episodes into Level 1 and Level 2 AH, we noticed that women are prone to Level 2 AH, which was in accordance with a prospective study by Zhang et al. [27]. The exact reason was unclear. As such, a larger sample size is needed in the future to verify the relationship between gender and AH.

Prior research found that lower HbA1c was an independent predictor of AH [28, 30, 31]; this was also highly salient in the current cohort. Currently, HbA1c is known to be the most used parameter to assess glycemic control and important index in the treatment of hyperglycemia, which has been used as the primary endpoint for many CGM studies [23]. However, new data support the need to devote attention to TIR and TAR, for a comprehensive evaluation of glycemic control among the diabetes population [12, 23, 32]. Indeed, some clinicians may choose to target the reduction of the TAR and minimize hypoglycemia, thereby arriving at more time in the target range. Whereas, recent recommendations from an international consensus on TIR emphasized that targets should be individualized and each 5% increase in TIR correlated with clinically meaningful benefits [23]. For the present study, in terms of lower HbA1c levels and higher percentages of TIR, the AH group seemed to had better blood glucose control than the non-AH group, but when integrated with MAGE, SD, and TAR, the higher glucose variability (GV) of the AH group is noteworthy. Accordingly, given the substantially high risk of AH in T2DM outpatients, clinicians should be vigilant in preventing hypoglycemia in such populations and avoid aggressively attempting to achieve near-normal glucose or HbA1c levels in whom the risks of lower glycemic targets may far outweigh the potential benefits on diabetic complications. So far, SD, MAGE, and MBG measured by CGMS, as key metrics for GV, has been confirmed to be closely correlated with hypoglycemia events [22, 33–35]. Providing unprecedented access to a range of new indicators of glucose control, CGMS, which would help clinicians and patients with diabetes to overcome the limitations of HbA1c and SMBG, should be brought into the routine management of diabetes to facilitate prompt therapy adjustment.

Our study had several strengths. Firstly, compared with similar domestic studies, the sample size of our study is larger. Secondly, after installing the CGMS device, all the participants returned home without any changes of routine life, making the risks of AH highly reliable. Besides, several weaknesses should be noted. First, our CGM data only covered three days of glucose metrics, which might underestimate the incidence of AH. Second, since we did not collect data of other possible causes of hypoglycemia, such as alcohol consumption and physical activities, it is impossible to determine whether these factors play a role in the occurrence of AH in T2DM. Finally, our study is a retrospective study, which has recalling bias. Therefore, further large samples of prospective studies are needed to consolidate the conclusions of this study, including long-term follow-up of the hypoglycemic outpatients after treatment and lifestyle adjustments.

## Conclusions

In summary, a high incidence of AH, of which NAH accounted for the main part, was observed in T2DM outpatients who did not receive intensive therapy. Relative relax HbA1c targets should be considered for patients who are prone to AH. We suggest CGMS should be intermittently used in the routine management of T2DM outpatients to timely capture hypoglycemia and minimize hypoglycemia exposure to benefit from better overall glucose control.

## Supplementary Information

**Additional file 1: Table S1.** Analysis of influencing factors of AH, Level 1 AH, and Level 2 AH by univariate logistic regression model.

## Data Availability

The datasets used during the current study are available from the corresponding author on reasonable request.

## Abbreviations

AH: Asymptomatic hypoglycemia
T2DM: Type 2 diabetes
CGMS: Continuous glucose monitoring system
BG: Blood glucose
NAH: Nocturnal asymptomatic hypoglycemia
DAH: Daytime asymptomatic hypoglycemia
NAH*: Asymptomatic hypoglycemia occurred exclusively at night
DAH*: Asymptomatic hypoglycemia occurred exclusively in the daytime
HbA1c: Glycated hemoglobin A1c
SBP: Systolic blood pressure
DBP: Diastolic blood pressure
DPP4i: Dipeptidylpeptidase-4 inhibitors
DCCT: Diabetes Control and Complications Trial
SMBG: Self-monitoring blood glucose
T1DM: Type 1 diabetes
SD: Glucose standard deviation
MBG: Mean blood glucose
MAGE: Mean amplitude of glycemic excursions
TIR: Time in range
TBR: Time below range
TAR: Time above range
CP: C-Peptide
GV: Glucose variability
