# Supplementary material for: Hypoglycemia unawareness identified by continuous glucose monitoring system is frequent in outpatients with type 2 diabetes without receiving intensive therapeutic interventions
Source: Diabetol Metab Syndr. 2022 Nov 28;14:180. doi: 10.1186/s13098-022-00959-x (PMC9703673; doi:10.1186/s13098-022-00959-x)
Supplement: Supplementary file 1 — Additional file 1: Table S1. Analysis of influencing factors of AH, Level 1 AH, and Level 2 AH by univariate logistic regression model. [file 13098_2022_959_MOESM1_ESM.docx]

**Hypoglycemia unawareness identified by continuous glucose monitoring system is frequent in outpatients with type 2 diabetes without receiving intensive therapeutic interventions**

Bingkun Huang1†, Qiuhui Jiang1,2†, Ting Wu1,2, Qingbao Shen1, Wengui Wang1, Shoubi Wang1,3, Yinxiang Huang1, Shunhua Wang1, Peiying Huang1, Mingzhu Lin1, Xiulin Shi1*, Xuejun Li 1*

**Affiliations:**

1. Department of Endocrinology and Diabetes, Xiamen Diabetes Institute, Xiamen Clinical Medical Center for Endocrine and Metabolic Diseases, Xiamen Diabetes Prevention and Treatment Center, Fujian Key Laboratory of Diabetes Translational Medicine, The First Affiliated Hospital of Xiamen University, School of Medicine, Xiamen University, Xiamen, China;

2. The Third Clinical Medical College of Fujian Medical University, Fujian, China;

3. Fujian Provincial Key Laboratory of Ophthalmology and Visual Science, Eye Institute of Xiamen University, School of Medicine, Xiamen University, Xiamen, China

***Correspondence:**

Dr Xuejun Li; xmlixuejun@163.com.

Dr Xiulin Shi [shixiulin2002@163.com](mailto:shixiulin2002@163.com)

†These authors contributed equally to this work and share first authorship.

**Supplementary Table S1.** Analysis of influencing factors of AH, Level 1 AH, and Level 2 AH by univariate logistic regression model.

|  |  |  |  |  |  | Univariate analysis | | |  |  |  |  |
| --- | --- | --- | --- | --- | --- | --- | --- | --- | --- | --- | --- | --- |
|  |  | AH | |  |  | Level 1 AH | | |  | Level 2 AH | | |
| Predictor |  | OR (95%Cl) | P |  |  | OR (95%Cl) | | P |  |  | OR (95%Cl) | P |
| Sex |  | 0.781(0.507-1.202) | 0.261 |  |  | 1.237(0.752-2.035) | | 0.402 |  |  | 0.468(0.258-0.849) | **0.013** |
| Age |  | 0.982(0.967-0.997) | **0.020** |  |  | 1.006(0.988-1.023) | | 0.536 |  |  | 0.959(0.939-0.980) | **<0.001** |
| Duration |  | 1.004(0.971-1.038) | 0.819 |  |  | 0.993(0.955-1.032) | | 0.717 |  |  | 1.017(0.973-1.063) | 0.456 |
| Metformin |  | 0.782(0.479-1.277) | 0.325 |  |  | 1.115(0.647-1.920) | | 0.695 |  |  | 0.496(0.231-1.066) | 0.072 |
| DPP-4i |  | 0.888(0.493-1.601) | 0.693 |  |  | 1.901(1.030-3.508) | | **0.040** |  |  | 0.084(0.011-0.623) | **0.015** |
| α-GI |  | 0.819(0.466-1.438) | 0.487 |  |  | 1.245(0.675-2.294) | | 0.483 |  |  | 0.420(0.160-1.105) | 0.079 |
| SU |  | 1.113(0.666-1.859) | 0.682 |  |  | | 1.453(0.827-2.554) | 0.194 |  |  | 0.679(0.316-1.460) | 0.322 |
| HbA1c |  | 0.820(0.695-0.967) | **0.018** |  |  | 0.756(0.610-0.935) | | **0.010** |  |  | 0.985(0.803-1.207) | 0.881 |
| Insulin |  | 1.250(0.802-1.949) | 0.325 |  |  | 0.798(0.477-1.334) | | 0.389 |  |  | 2.078(1.133-3.814) | **0.018** |
| SBP |  | 1.003(0.989-1.018) | 0.682 |  |  | 1.013(0.997-1.030) | | 0.102 |  |  | 0.985(0.963-1.006) | 0.158 |
| DBP |  | 0.991(0.967-1.014) | 0.429 |  |  | 1.002(0.976-1.029) | | 0.885 |  |  | 0.979(0.947-1.012) | 0.213 |
| BMI |  | 0.937(0.868-1.010) | 0.090 |  |  | 0.926(0.849-1.010) | | 0.084 |  |  | 0.988(0.892-1.095) | 0.821 |

OR, odds ratio; CI, confidence interval. DPP-4i, Dipeptidylpeptidase-4 inhibitors; α-GI, alpha-glucosidase inhibitors; SU, sulfonylurea; HbA1c, glycated hemoglobin; SBP, systolic blood pressure; DBP, diastolic blood pressure; BMI, body mass index.
